# Associations between Intake of Dietary Flavonoids and 10-Year Incidence of Age-Related Hearing Loss

**DOI:** 10.3390/nu12113297

**Published:** 2020-10-28

**Authors:** Bamini Gopinath, Catherine M. McMahon, Joshua R. Lewis, Nicola P. Bondonno, Catherine P. Bondonno, George Burlutsky, Jonathan M. Hodgson, Paul Mitchell

**Affiliations:** 1 Department of Linguistics, Macquarie University Hearing, Macquarie University, Balaclava Rd, Macquarie Park, NSW 2109, Australia; cath.mcmahon@mq.edu.au; 2 Department of Ophthalmology and Westmead Institute for Medical Research, Centre for Vision Research, University of Sydney, Camperdown, NSW 2006, Australia; george.burlutsky@sydney.edu.au (G.B.); paul.mitchell@sydney.edu.au (P.M.); 3 School of Public Health, Centre for Kidney Research, Westmead, University of Sydney, Camperdown, NSW 2006, Australia; joshua.lewis@ecu.edu.au; 4 School of Medical and Health Sciences, Edith Cowan University, Perth, 270 Joondalup Dr, Joondalup, WA 6027, Australia; n.bondonno@ecu.edu.au (N.P.B.); c.bondonno@ecu.edu.au (C.P.B.); jonathan.hodgson@ecu.edu.au (J.M.H.); 5 School of Medicine, University of Western Australia, 35 Stirling Highway, Perth, WA 6009, Australia

**Keywords:** age-related hearing loss, Blue Mountains Hearing Study, flavonoids, isoflavone, incidence

## Abstract

Dietary flavonoids are vasoactive phytochemicals with promising anti-inflammatory properties. We aimed to assess the associations between baseline intakes of six commonly consumed flavonoid subclasses and 10-year incidence of age-related hearing loss. At baseline, 1691 participants aged 50+ years had information on dietary intakes and hearing status. Hearing loss was defined as the pure-tone average of frequencies 0.5, 1.0, 2.0, and 4.0 kHz > 25 dB hearing level (HL). Dietary data were collected through a semi-quantitative food frequency questionnaire. The flavonoid content of foods was estimated using US databases. During the 10-year follow-up, 260 (31.6%) new cases of hearing loss (incident) were observed. After multivariable adjustment, participants in the fourth versus first quartile (reference group) of intake of dietary isoflavone had 36% lower risk of incident hearing loss after 10 years: odds ratios (OR) 0.64 (95% confidence intervals, CI, 0.42–0.99); *p-value for trend* = 0.03. Nonsignificant associations were observed between the other five flavonoid subclasses and 10-year incidence of hearing loss. Our findings do not support the hypothesis that the intake of dietary flavonoids protect against long-term risk of hearing loss. The association with isoflavone intake needs to be confirmed by other population-based studies.

## 1. Introduction

Age-related hearing loss is a decrease in hearing ability that occurs with age and is projected to be within the top 15 leading causes of the burden of disease by 2030 [1]. The communication problems associated with age-related hearing loss can lead to reduced quality of life, depressive symptoms, cognitive decline, functional disability, and reduced life expectancy [2,3,4,5]. Age-related hearing loss is a condition that involves several modifiable and nonmodifiable risk factors such as noise exposure, smoking, and specific genetic risk mutations [6]. Dietary factors are suspected to play a pivotal, yet poorly, defined role in hearing loss among older adults [7].

Reactive oxygen metabolites and inflammatory processes may cause cochlear damage [8]. Therefore, it has been hypothesized that dietary antioxidants and anti-inflammatory components could help to preserve auditory cells, e.g., spiral ganglion. This could minimize the hearing loss linked to aging [9]. Flavonoids, a class of bioactive compounds, might have a role to play in reducing the risk of hearing loss, as they have been shown to have local antioxidant and anti-inflammatory activities and enhance vascular health [10,11]. Preclinical models suggest that flavonoids, such as epicatechin, may reduce hearing loss resulting from treatment with the chemotherapeutic drug cisplatin by minimizing the excessive production of reactive oxygen species and proinflammatory mediators [12,13,14]. Further, evidence shows that flavonoids positively impact vascular health through improved endothelial function [15] and thus, these compounds could help to maintain the integrity of the capillary bed within the cochlea [16].

However, to our best knowledge, no large prospective cohort study has assessed the temporal association between the intake of dietary flavonoids and age-related hearing loss. Therefore, we used a large, representative, population-based cohort to investigate longitudinal associations between six common flavonoid subclasses (flavonols, flavan-3-ols, flavones, flavanones, anthocyanins, and isoflavones) and the 10-year incidence of hearing loss among adults aged 50+ years.

## 2. Methods

### 2.1. Study Participants

The Blue Mountains Hearing Study (BMHS) is a survey of age-related hearing loss from 1997 to 2004 among participants of the Blue Mountains Eye Study (BMES) cohort as previously described [17]. Briefly, hearing was measured from the second wave, i.e., BMES-2 (in 1997–1999) onwards or BMHS, with 2956 participants aged ≥50 y undergoing audiometric testing and, subsequently, participants were followed up over a period of 10 years, until 2007–2009. The study was approved by the Human Research Ethics Committee (HREC) of the University of Sydney (HREC approval number-9826) and was conducted adhering to the tenets of the Helsinki Declaration. Signed, informed consent was obtained from all the participants at each examination.

### 2.2. Hearing Assessment

We asked questions on family history of hearing loss and occupational noise exposure: ‘Have you ever worked in a noisy industry or noisy farm environment?’ Pure-tone audiometry was performed by audiologists in sound-treated booths, as previously described [2,3,4,5]. We defined any level of hearing loss as pure tone audiometry (PTA)_0.5-4KHz_ > 25 dB hearing level (HL) in the better of the two ears, i.e., bilateral hearing loss [18].

Risk of incident bilateral hearing loss during the 10 years (from BMES-2 to BMES-4) was considered among participants who had PTA_0.5-4KHz_ in the better ear of ≤25 dB HL at BMES-2 or BMES-3. Incident bilateral hearing loss was defined as a PTA_0.5-4KHz_ >25 dB HL in the better ear at the 5- or 10-year follow-up examination among participants without hearing impairment.

### 2.3. Measuring Dietary Flavonoid Intake

A modified, 145-item, self-administered food frequency questionnaire (FFQ) was used to collect information on dietary intakes [19]. The typical frequency of consuming individual food items over the year was determined and these items were then classified using the same approach as in the 1995 Australian National Nutrition Survey [20]. Flavonoid content of foods in the FFQ were calculated from several US databases [21,22,23].

For each food, we assessed the intake of each individual flavonoid compound found in the food and determined the sum of measured flavonoids for each flavonoid class, as previously described [24]. The flavan-3-ol content of foods was considered to represent the average of total flavan-3-ol and proanthocyanidin monomer contents. For foods where only the flavan-3-ol or proanthocyanidin monomer content was available, the single value provided was used to represent the flavan-3-ol content. The proanthocyanidin content of foods was calculated by summing the proanthocyanidin dimers, trimers, 4–6 mers, 7–10 mers, and polymers. Intakes of flavonoid classes (in mg/d) were determined by multiplying the estimated intake (g edible portion/d) from the FFQ, with the flavonoid class content of each food item on the questionnaire. Estimations for some foods were made using generic recipes found online [25,26].

### 2.4. Statistical Analysis

SAS statistical software (SAS Institute, Cary, North Carolina, USA) version 9.4 was used for analyses. Associations between dietary flavonoid intakes (study factor) and 10-year incidence of age-related hearing loss (study outcome) were examined in discrete logistic regression models. Regression analysis first adjusted for age and sex and then for covariates that were found to be associated with incidence of age-related hearing loss in the BMHS cohort: Family history of hearing loss and exposure to noise at work [27]. Findings from all analyses are expressed as adjusted odds ratios (OR) with 95% confidence intervals (CI).

### 2.5. Sources of Support

The Blue Mountains Eye and Hearing Studies were supported by the Australian National Health and Medical Research Council (Grant Nos. 974159, 991407, 211069, 262120).

## 3. Results

Of the 2956 participants who had audiometric testing performed at BMES-2 (i.e., baseline BMHS), 1691 had complete information on dietary intakes. Of these 1691 baseline participants, 647 participants had bilateral hearing loss and were excluded from subsequent incidence analysis. Those who had hearing loss versus those who had normal hearing function at baseline were more likely to be male, older, and have a family history of hearing loss (Table 1). Over the 10-year follow-up, 260 (31.6%) new cases of hearing loss (incident) were observed.

## 4. Discussion

The current study findings do not provide evidence that total flavonoid intake and the intakes of specific flavonoid subclasses influence the risk of age-related hearing loss. However, we observed a modest inverse association between the intakes of isoflavones and 10-year incidence of hearing loss, indicating a potential protective effect against the development of hearing loss.

Although we demonstrated an association between a greater intake of dietary isoflavones and a lower risk of age-related hearing loss, we were unable to determine whether the observed association indicated a benefit of the isolated bioactive, per se, or whether it is a signal of the bioactives working together with the co-occurring phytochemicals within isoflavone-containing foods [28]. We also caution that this observed finding could be due to chance. Isoflavones are found in minimal quantities in vegetables and grains; their only rich sources include soy products. Indeed, isoflavone intakes in this cohort were estimated to be very low (mean intake of 1.29 mg/day). However, soy and isoflavone intakes were likely to be underestimated in the BMHS as there were no questions about soy products, such as tofu and soy milk, or products with added soy protein such as specific breads. In Australian populations using FFQs designed to capture soy and isoflavone intakes, estimated intakes usually average 3–15 mg/d [29,30], whereas in Asian populations intakes are often in the range of 25–50 mg/d [28].

Nevertheless, we can posit a potential mechanism by which intake of isoflavones could protect against age-related hearing loss. For instance, isoflavones are considered to be ‘phytoestrogens’ with multiple potential cardioprotective benefits. They are structurally similar to estradiol and may reduce low-density lipoprotein cholesterol, induce nitric oxide production, and improve vascular reactivity [31,32]. Recently, Ma et al. [33] demonstrated in three prospective cohorts of US men and women with more than two decades of follow-up that a higher isoflavone intake was associated with a moderately lower risk of coronary heart disease. Atherosclerotic changes underlie both age-related hearing loss and cardiovascular disease [34]. Specifically, an atherosclerotic inflammatory change that causes reduced blood supply to the inner ear [35] could, in turn, lead to a decrease in the supply or delivery of oxygen and nutrients and removal of waste material, rendering the inner ear to be more prone to damage from other factors such as noise exposure and aging itself [36]. Thus, a mechanistic link between greater isoflavone intake and a lower risk of hearing loss could be mediated via improvements in cardiovascular health. The lack of evidence to support an association for other flavonoid classes with hearing loss would not support many of these proposed mechanisms. If the association of isoflavones with age-related hearing loss were causal via antioxidant, anti-inflammatory, or vasoactive effects, then similar associations would be expected for other flavonoid classes. Our analysis provides no evidence for this. However, the observed associations may be consistent with a phytoestrogen-linked pathway caused by either the parent isoflavones or their metabolites.

Overall, our results do not support a protective effect of most of the flavonoid classes against age-related hearing loss. The largely nonsignificant findings regarding total flavonoids and flavonoid subclasses could be due to the oral bioavailability of flavonoids being constrained by poor intrinsic transmembrane diffusion features [37]. In addition, with the exception of isoflavone metabolites [38,39,40], the activity of most flavonoid metabolites is not well established [37]. Additional studies are, therefore, needed to investigate whether systemic administration of flavonoids will result in higher and effective concentrations of the parent flavonoids in the inner ear and cochlear at much lower doses and whether this would translate to a reduced risk of age-related hearing loss in the longer term.

The strengths of our study include its representative sample of older adults with relatively high participation rates, minimizing selection bias. Furthermore, we used standardized, audiometric testing to measure hearing sensitivity and a validated food questionnaire to collect dietary information. Additionally, study participants were not aware of the study question and our dietary data were collected before detection of incident hearing loss, reducing indication bias arising from behavioral change after the hearing loss was diagnosed. However, this study is not without its limitations. First, flavonoid content of foods was solely estimated using US-based databases and, hence, this approach might have discounted any variation in the flavonoid content of Australian foods [26]. We also cannot disregard the effect of residual confounding from covariates that were not assessed in our study (e.g., inflammatory markers).

In summary, our study findings suggest that intakes of dietary flavonoid subclasses and compounds are unlikely to have a role in preserving healthy auditory function among older adults. However, we found that a higher intake of dietary isoflavones reduced the risk of incident hearing loss by over a third. Further large, prospective studies are now warranted to verify our findings and to establish whether aural benefits result from treatment with specific dietary flavonoids.

## Figures and Tables

**Table 1:** Characteristics of Blue Mountains Hearing Study participants stratified by hearing status at baseline.

Characteristics	No Hearing Loss (*n* = 1044)	Any Hearing Loss (*n* = 647)	*p*-Value
Men, *n* (%)	405 (38.8)	318 (49.2)	<0.0001
Age, *mean(std)*	66.6 (7.1)	74.2 (7.9)	<0.0001
Tertiary educational qualifications, *n* (%)	656 (65.6)	353 (56.6)	0.0003
Exposed to noise at work, *n* (%)	360 (34.7)	273 (42.3)	0.002
Family history of hearing loss, *n* (%)	432 (41.4)	296 (45.8)	0.08
Current smoking, *n* (%)	92 (8.8)	47 (7.3)	0.267
BMI(kg/m^2^), *mean(std)*	27.5 (4.5)	26.9 (4.4)	0.0064
History of hypertension, *n* (%)	541 (52.0)	375 (58.1)	0.0134
History of angina, *n* (%)	96 (9.2)	105 (16.4)	<0.0001
History of myocardial infarction, *n* (%)	75 (7.2)	66 (10.3)	0.026
History of stroke, *n* (%)	36 (3.5)	48 (7.5)	0.0002
Type 2 diabetes, *n* (%)	89 (8.5)	83 (12.8)	0.0045
Use of statins, *n* (%)	101 (9.7)	63 (9.7)	0.97

After adjusting for age, sex, noise exposure, and family history of hearing loss, nonsignificant associations were observed between intake of total flavonoids, flavonol, flavanone, flavan-3-ol, flavone, anthocyanidin, and proanthocyanidin with incident hearing loss (Table 2). Participants in the fourth versus first quartile (reference group) of intake of dietary isoflavone had 36% lower risk of incident hearing loss after 10 years: Multivariable-adjusted odds ratios (OR) 0.64 (95% confidence intervals, CI, 0.42-0.99); *p-value for trend* = 0.03 (Table 2). Given the significant associations between isoflavone, we looked at the key individual compounds that constitute this flavonoid subclass, that is, dietary intake of genistein and daidzein. Participants in the highest versus lowest quartile of intake of genistein at baseline had 37% reduced risk of incident hearing loss 10 years later (*P_trend_* = 0.03): Multivariable-adjusted OR 0.63 (95% CI 0.41–0.96). However, no significant associations were observed between baseline intake of daidzein and risk of incident hearing loss–multivariable-adjusted *P_trend_* = 0.10.

**Table 2:** Multivariable adjusted associations between baseline flavonoid intake and 10-year incidence of bilateral age-related hearing loss (>25 dB hearing level {HL}).

	Multivariable-Adjusted *
Flavonoids (mg/day)	Odds Ratio (95% Confidence Intervals)
All flavonoids	
1st quartile (≤310.3)	1.0 (reference)
2nd quartile (311.9–795.5)	1.09 (0.70–1.69)
3rd quartile (795.8–1197.7)	1.19 (0.78–1.84)
4th quartile (≥1200.9)	1.25 (0.82–1.92)
*P* for trend	0.27
Total flavonol	
1st quartile (≤17.8)	1.0 (reference)
2nd quartile (17.8–33.9)	1.02 (0.66–1.58)
3rd quartile (34.0–45.4)	0.97 (0.63–1.50)
4th quartile (≥45.5)	1.19 (0.78–1.82)
*P* for trend	0.48
Total flavanone	
1st quartile (≤6.5)	1.0 (reference)
2nd quartile (6.6–21.9)	0.84 (0.55–1.29)
3rd quartile (22.1–44.6)	0.89 (0.59–1.35)
4th quartile (≥44.7)	0.87 (0.56–1.35)
*P* for trend	0.70
Total flavan-3-ol	
1st quartile (≤238.0)	1.0 (reference)
2nd quartile (238.3–729.3)	1.08 (0.69–1.68)
3rd quartile (729.4–1146.7)	1.32 (0.86–2.02)
4th quartile (≥1146.7)	1.35 (0.88–2.07)
*P* for trend	0.11
Total anthocyanidin	
1st quartile (≤3.5)	1.0 (reference)
2nd quartile (3.5–6.5)	1.07 (0.68–1.70)
3rd quartile (6.5–16.7)	1.38 (0.89–2.14)
4th quartile (≥16.9)	0.96 (0.61–1.52)
*P* for trend	0.42
Proanthocyanidin	
1st quartile (≤71.9)	1.0 (reference)
2nd quartile (71.9–116.6)	1.30 (0.83–2.04)
3rd quartile (117.2–172.4)	1.06 (0.69–1.65)
4th quartile (≥172.5)	1.32 (0.85–2.05)
*P* for trend	0.39
Total flavone	
1st quartile (≤0.61)	1.0 (reference)
2nd quartile (0.62–1.02)	1.02 (0.67–1.55)
3rd quartile (1.02–1.56)	0.66 (0.42–1.03)
4th quartile (≥1.57)	0.71 (0.45–1.12)
*P* for trend	0.06
Isoflavone	
1st quartile (≤0.73)	1.0 (reference)
2nd quartile (0.73–1.04)	0.88 (0.58–1.34)
3rd quartile (1.05–1.49)	0.67 (0.43–1.03)
4th quartile (≥1.49)	0.64 (0.42–0.99)
*P* for trend	0.03

* Adjusted for age, sex, noisy work environment, and family history of hearing loss.
